# The Value of Remote Vital Signs Monitoring in Detecting Clinical Deterioration in Patients in Hospital at Home Programs or Postacute Medical Patients in the Community: Systematic Review

**DOI:** 10.2196/64753

**Published:** 2025-05-26

**Authors:** Su-Ann Cheng, Shijie Ian Tan, Samuel Li Earn Goh, Stephanie Q Ko

**Affiliations:** 1 Department of Medicine National University Hospital National University Health System Singapore Singapore; 2 Division of Advanced Internal Medicine National University Hospital National University Health System Singapore Singapore; 3 NUHS@Home National University Health System Singapore Singapore

**Keywords:** hospital at home, remote vital signs monitoring, postacute care, hospital discharge, early supported discharge, admission avoidance, ambulatory monitoring, transitional care, post hospital, care delivery, vital signs, blood pressure, heart rate, temperature, oxygen saturation, respiratory rate, pulse rate, PRISMA

## Abstract

**Background:**

Vital signs monitoring (VSM) is used in clinical acuity scoring systems (APACHE [Acute Physiology and Chronic Health Evaluation], NEWS2 [National Early Warning Score 2], and SOFA [Sequential Organ Failure Assessment]) to predict patient outcomes for early intervention. Current technological advances enable convenient remote VSM. While the role of VSM for ill, hospital ward–treated patients is clear, its role in the community for acutely ill patients in the hospital at home (HAH) or postacute setting (patients who have just been discharged from an acute hospital stay and at increased risk of deterioration) is less well defined.

**Objective:**

We assessed the efficacy of remote VSM for patients in the HAH or postacute setting.

**Methods:**

This systematic review adhered to the PRISMA (Preferred Reporting Items for Systematic Reviews and Meta-Analyses) methodology. We searched studies in PubMed (MEDLINE), Embase, and Scopus. Studies focused on the postacute phase were included, as only 2 case series addressed the HAH setting. Risk of bias (ROB) was evaluated using the Cochrane Risk of Bias Tool for randomized controlled trials (RCTs), the Newcastle-Ottawa scale for observational studies, and the case methods outlined by Murad et al for case reports. The GRADE (Grading Recommendations Assessment, Development, and Evaluation) framework was used to assess the certainty of evidence. Outcomes of interest included hospital readmissions, mortality, patient satisfaction, and compliance. Risk ratios (RR) were used to measure effect sizes for readmission and mortality, with patient satisfaction and compliance reported descriptively.

**Results:**

The search yielded 5851 records, with 28 studies meeting eligibility criteria (8 RCTs, 7 cohort studies, and 13 case series). Two focused on HAH, while 26 studies addressed the postacute phase. Nineteen studies looked at heart failure, 3 studied respiratory conditions, and 6 studies studied other conditions. Meta-analysis was conducted with 6 studies looking at hospital readmission within 60 days and 4 studies at mortality within 30 days. Readmissions did not significantly decrease (RR 0.81, 95% CI 0.61-1.09; *P*=.16). Significant heterogeneity was observed for readmissions (*I*^2^=58%). Conversely, mortality reduced significantly (RR 0.65, 95% CI 0.42-0.99; *P*=.04). There was no significant heterogeneity in mortality (*I*^2^=0%). There was high heterogeneity in the study populations, interventions, and outcomes measured. Many studies were of poor quality, with 50% (4/8) of RCTs exhibiting a high ROB. The certainty of evidence for both readmission and mortality was very low.

**Conclusions:**

Published data on the effects of remote VSM in acutely ill patients at home remains scarce. Future studies evaluating all common vital signs (heart rate, blood pressure, oxygen saturation, and temperature) with consistent monitoring frequencies and clear intervention protocols to better understand how to integrate remote VSM into HAH programs are needed.

**Trial Registration:**

PROSPERO CRD42023388827; https://www.crd.york.ac.uk/PROSPERO/view/CRD42023388827

## Introduction

Vital signs monitoring (VSM) is a core component of clinical practice. It holds independent predictive value for mortality, has been validated to provide early warning signs [1], and is an essential component of many scoring systems predicting clinical deterioration [2]. VSM is typically conducted for hospitalized patients every 4-8 hours with only a minority of patients receiving less frequent monitoring [3]. With the advancements in medical and commercial technology, VSM can now be easily performed remotely at home. Concurrently, advancements in videoconferencing, improved internet connectivity, and Bluetooth technology have streamlined telehealth conferencing [4]. The COVID-19 pandemic further accelerated the adoption of telehealth [5] and remote VSM at home.

While the role of VSM in acute illnesses treated in hospital wards is clear, its role in acute illnesses treated at home is less well-defined. Hospital at home (HAH) is a care delivery model that furnishes acute-level hospital care and services within patients’ homes [6]. HAH has been shown to be a safe alternative to inpatient care while reducing health care expenses [7] and improving patient satisfaction [8]. It has been suggested that, similar to the inpatient wards, VSM in HAH programs could improve patient safety and increase eligibility for patients with moderate acuity, as well as those from remote or regional areas [9]. However, there are significant differences between patients in HAH programs compared with patients hospitalized in a ward. Patients admitted to HAH are usually preselected to be more stable and at lower risk of deterioration [10]. Patients in HAH programs also tend to be ambulant and more active at home [11]. In addition, VSM in hospital wards is conducted by medically trained personnel, compared with patient or caregiver-initiated VSM in HAH. Questions also remain regarding the optimal modality and frequency of remote VSM with many HAH programs either not incorporating remote VSM or using it with significant variation in terms of frequency and modality [12].

This review aims to investigate the effectiveness of remote VSM on patients in HAH programs. Due to the anticipated paucity of data specifically in the HAH setting, we also included studies from the postacute phase, where patients are in the community but remain at risk of deterioration.

## Methods

This systematic review was conducted in accordance with the PRISMA (Preferred Reporting Items for Systematic Reviews and Meta-Analyses) methodology, which can be found in the Multimedia Appendix 1. It was registered in PROSPERO (International Prospective Register of Systematic Reviews; CRD42023388827) on January 17, 2023 [13].

### Search Strategy and Eligibility Criteria

A literature search was performed on January 16, 2023, using a search strategy approved by all authors in the following databases: PubMed (MEDLINE), Embase, and Scopus. The search strategy was developed through secondary research, with input from a librarian at the National University of Singapore to refine the approach. Keywords included “hospital discharge,” “transitional care,” “postadmission,” “after admission,” “postdischarge,” “after hospital,” “posthospital,” “postacute,” “hospital at home,” “hospital in the home,” “home hospital,” “early supported discharge,” “admission avoidance,” “ambulatory monitoring,” “vital signs,” “blood pressure,” “heart rate,” “temperature,” “oxygen saturation,” “respiratory rate,” and “pulse rate” with publications limited to those from 2012 and in English. The full search strategies can be found in the Multimedia Appendix 2. Duplicates were removed via CADIMA (Cadima), a free web tool for systematic reviews [14].

Studies focused on the postacute phase, where patients are in the community but at risk of deterioration, were included because of the scarcity of studies specific to the HAH setting. Studies evaluating remote home monitoring of at least one vital parameter were included, as there were few studies that recorded all the common vital parameters. All study designs were considered eligible for inclusion. Abstracts without full-text publications were also included due to the limited availability of full-text publications. The outcomes of interest were limited to emergency department representations, hospital readmissions or return to hospital (RTH), which reflect the likelihood of patients needing additional medical care or hospitalization over an extended period, along with mortality, as these were the key clinical outcomes assessed in most HAH studies.

Studies including surgical patients were excluded to minimize heterogeneity between studies, as surgical patients tend to have higher immediate risks of adverse effects, infections, and perioperative complications [15,16]. Studies not reporting on VSM, even if telehealth was used, were not included to ensure that our evaluation was not confounded by the effects of telehealth.

Therefore, to be included in this review, studies had to meet the following criteria:

1. Patients admitted to an HAH program or immediately following discharge from an acute admission.
2. Patients aged 21 years and older.
3. Remote home monitoring of at least one vital parameter (body temperature, respiratory rate, heart rate, blood pressure, or oxygen saturation) in a community setting.
4. Outcomes of emergency department representations, hospital readmissions, or mortality.

Studies were excluded if they met with any of the following criteria:

1. Involved patients only received care in the acute hospital setting.
2. Included a pediatric cohort (participants younger than 21 years).
3. Encompassed patients recovering from surgeries.
4. No VSM, even if telehealth was used post discharge.
5. They were not published in English.

All abstracts were independently reviewed by 2 authors (TSI and CS), after which the full text versions of potentially eligible studies were accessed for eligibility before data extraction. The reference lists of included articles and reviews were manually searched, and relevant studies were added for full-text review. Any discrepancies between authors on the article were resolved through consensus among all 3 authors (TSI, CS, and SQK).

### Data Extraction

A total of 2 investigators (TSI and CS) independently conducted data extraction using a standardized data collection form tailored for this review. Any inconsistencies were resolved through discussion, resulting in complete consensus. In cases of uncertainty or insufficient data, the original investigators were contacted via email for clarification. The following data were extracted: patient demographics, primary condition studied, vital sign parameters measured, method and transmission type used for VSM, and interventions used if abnormal VSM were detected. The outcomes of interest included readmission rates, mortality rates, patient satisfaction, and compliance. Effect size was measured using risk ratios for readmission and mortality rates, while patient satisfaction and compliance were assessed as described. Due to the paucity of studies, all studies which reported on readmission rates within 60 days were synthesized together. Similarly, all studies that reported on mortality within 30 days were synthesized together.

### Risk of Bias

Risk of bias was analyzed independently by TSI and CS using the Cochrane Risk of Bias Tool (ROB-2) quality assessment tool for RCTs [17], Newcastle-Ottawa scale for observational studies [18], and case methods described by Murad et al [19], for case reports. Discrepancies between authors on risk of bias were resolved by all 3 authors (TSI, CS, and SQK). Any discrepancies were resolved through consensus.

### Statistical Analysis

Estimates of readmission and mortality rates from randomized controlled trials (RCTs) and cohort studies were combined using risk ratios (RR). A random-effects model [20] was applied with the Mantel-Haenszel method, accounting for expected between-study differences. Heterogeneity was evaluated using the *I*^2^ statistic, with an *I*^2^ greater than 50% indicating substantial heterogeneity [21]. Publication bias was evaluated using funnel plots. All analyses were conducted with Review Manager Software (RevMan version 5.4; Cochrane Collaboration) [22]. Data not amenable to meta-analysis were described and synthesized narratively. Due to the small number of studies within each analysis, subgroup analysis and meta-regression were unable to be conducted.

### Certainty of Evidence

The GRADE (Grading Recommendations Assessment, Development, and Evaluation) framework was used to assess the certainty of evidence for both meta-analyses [23].

## Results

### Overview

**Figure 1:**
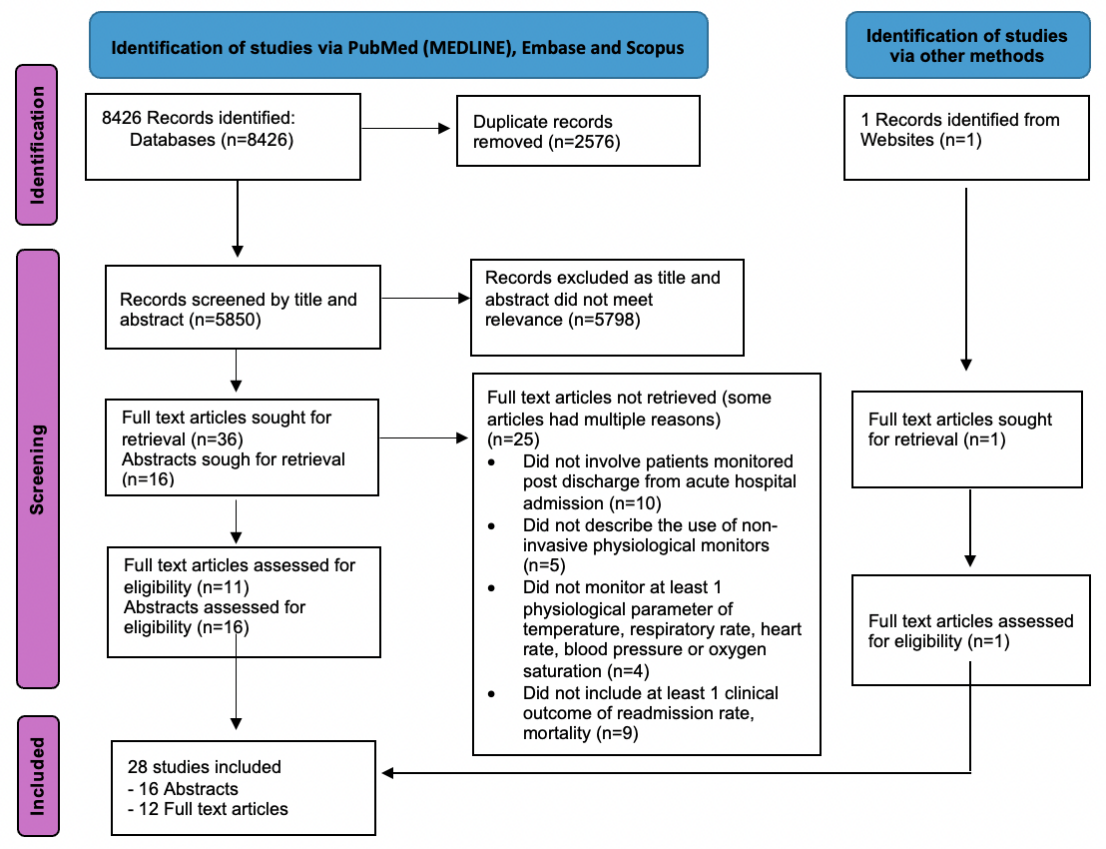
PRISMA flow diagram.

Figure 1 presents a flow diagram outlining the systemic search. Of the 8426 records initially identified, 2576 duplicates were excluded before screening. An additional 5797 records were excluded after screening by title and abstract. A total of 37 full text articles were screened. A total of 25 full texts were excluded with the most common reason for exclusion being that the publication did not involve patient monitored post discharge from acute hospital admissions. As a result, 16 abstracts were included given the small number of eligible full text articles (n=12). In total, 28 [24-51] articles (including 1 record identified from the website) were reviewed for data extraction.

A summary of the included studies [24-51] and their clinical outcomes can be found in the E Table S1-S2 in Multimedia Appendix 2. Around 16 (57%) studies [24,30,31,33,36-38,40-42, 44,46-49,51] were abstract only. There were 8 (29%) [24-31] RCTs, 7 (25%) [32-38] cohort studies, and 13 (46%) [39-51] case series. Notably, only 2 (7%) [40,51] studies, both of which were case series, examined VSM within HAH programs, while the remaining 26 (93%) studies [24-39,41-50] focused on patients in the postacute phase. Studies were grouped and reported based on study design.

The studies looked at a diverse range of medical conditions. Among the 28 [24-51] included articles, several focused on specific conditions. Heart failure was the most commonly studied condition, featured in 18 (64%) [24-26,28-34, 37-39,42-44,47,50] articles. Three (11 %) [27,36,46] examined multiple unspecified comorbidities, while 2 (7%) [45,48] articles focused on chronic obstructive pulmonary disease. Other conditions studied included acute coronary syndrome (n=1, 4%) [26], liver decompensation (n=1, 4%) [35], and pneumonia (n=1, 4%) [49], 1 (4%) study [40] focused on autologous stem cell transplantation, and 1 (4%) study [51] focused on Chimeric antigen receptor T-cell therapy. One (4%) [41] article did not provide information on the specific condition.

A variety of hospital readmission and mortality outcomes were reported across different time frames in the 28 [24-51] articles reviewed. The most commonly reported outcome was 30-day hospital readmission, which appeared in 13 (46%) studies [24-27,29,33,34,36,38,39,42,44,48]. Several studies also examined combined outcomes, including 30-day hospital readmission or 30-day mortality, mentioned in 2 studies (7%) [25,44], and 30-day hospital readmission or 6-month hospital readmission or 30-day mortality reported in 2 (7%) studies [26,29]. A total of 3 (11%) studies [34,43,50] reported 90-day hospital readmission and 2 (7%) studies [28,32] reported 12-month hospital readmission or mortality. Other time frames included 28-day (1, 4% studies) [45], 45-day (1, 4% studies) [30], 50-day and 170-day readmissions (1, 4% studies) [47], and 60-day readmission or mortality in 2 (7%) studies [31]. In addition, bed days saved per patient were reported in 1 (4%) study [40], and length of stay was mentioned in 1 (4%) study [46].

Smartphone apps with Bluetooth technology were used by 18 (64%) studies [24,26-33,35,38,39,41,42,45,47,50,51], while 8 (29%) studies [32,35,36,39,44,46,47,49] implemented wireless self-reporting (E Table S3 in Multimedia Appendix 2). Follow-up through smartphone apps was used by 22 (79%) studies [24-32,34,36,37,39-43,45-47,49,51], while 6 (21%) studies [33,35,36,38,44,46] used phone calls, and 2 (7%) studies [27,35] used video calls. Among these, 20 (71%) studies [25-31,33,35-38,40-45,47,51] measured blood pressure, 20 (71%) studies [25-33,35,36,39-41,44,45,47,48,50,51] measured heart rates, 16 (57%) studies [26-31,34-36,38,41,43-45,47,51] measured weight, 13 (46%) studies [25,27,28,33,37,40,41, 43-45,47,50,51] measured pulse oximetry, 1 (4%) study [5] measured blood glucose, and 5 (18%) studies [28,30,35,42,50] recorded electrocardiographs. In the case of abnormal results, 14 (50%) studies [24-31,33,38,44,45,47,50] sent prompts solely to nurses, 1 (4%) study [40] exclusively alerted physicians, and 5 (18%) studies [35,36,41,42,49] alerted both physicians and nurses.

### Risk of Bias

Regarding the quality of the studies, out of the 8 RCTs reviewed [1], 4 (50%) [24,25,30,31] exhibited a high risk of bias, whereas the other 4 (50%) [26-29] displayed some risk of bias (E Table S4 in Multimedia Appendix 2). Among the 7 cohort studies [32-38], 5 (71%) [32,34-36,38] were of good quality, 1 (14%) [37] was of poor quality, and 1 (14%) [33] was of fair quality (E Table S5 in Multimedia Appendix 2). Among the 13 case studies [39-51], based on a quality score out of 8, 4 (31%) studies [39,43,45,50] obtained a score of 5, 4 (31%) studies [40-42,48] obtained a score of 4, 4 (31%) studies [44,46,47,49] scored 3 points, and 1 (7%) study [51] scored 1 point (E Table S6 in Multimedia Appendix 2). There was no concern about reporting bias.

### Hospital Readmission

**Figure 2:**
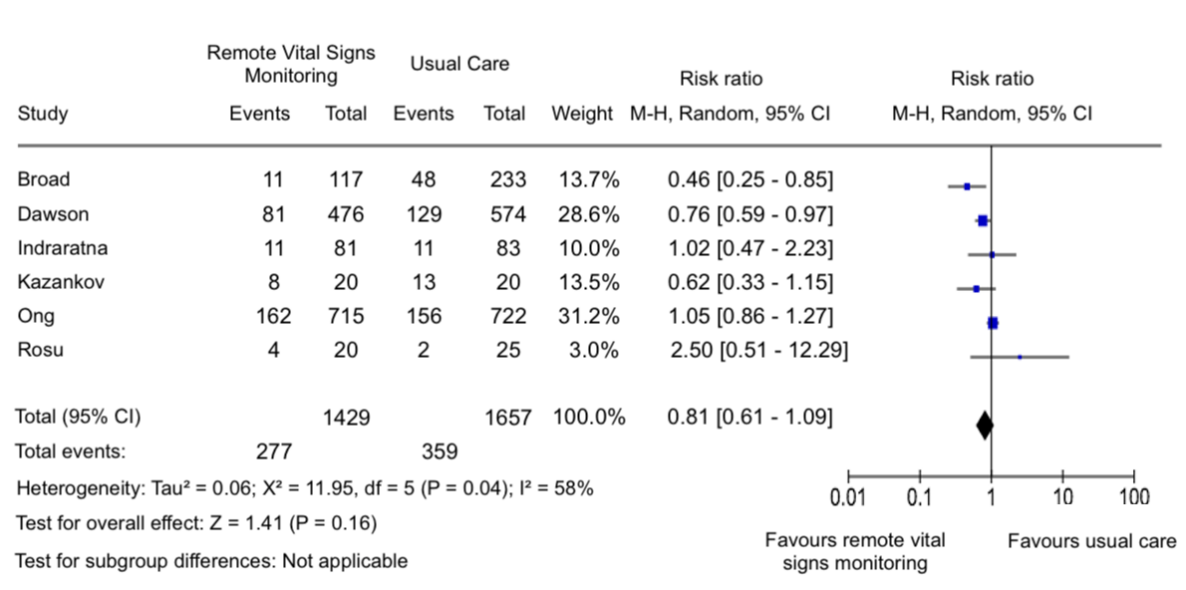
Forest plot of 6 [25,26,29,30,34,35] studies comparing remote vital signs monitoring against usual care on 60 day readmission.

Among 6 studies [25,26,29,30,34,35] that reported hospital readmission within 60 days, there was no significant reduction of hospital readmission within 60 days (risk ratio [RR] 0.81, 95% CI 0.61-1.09; Figure 2). Significant heterogeneity was observed for hospital readmissions within 60 days (*I*^2^=58%). Publication bias was observed for readmission in the funnel plot (E Figure S1 in Multimedia Appendix 2). The certainty of evidence (GRADE) is very low (E Table S5 in Multimedia Appendix 2).

### Mortality

**Figure 3:**
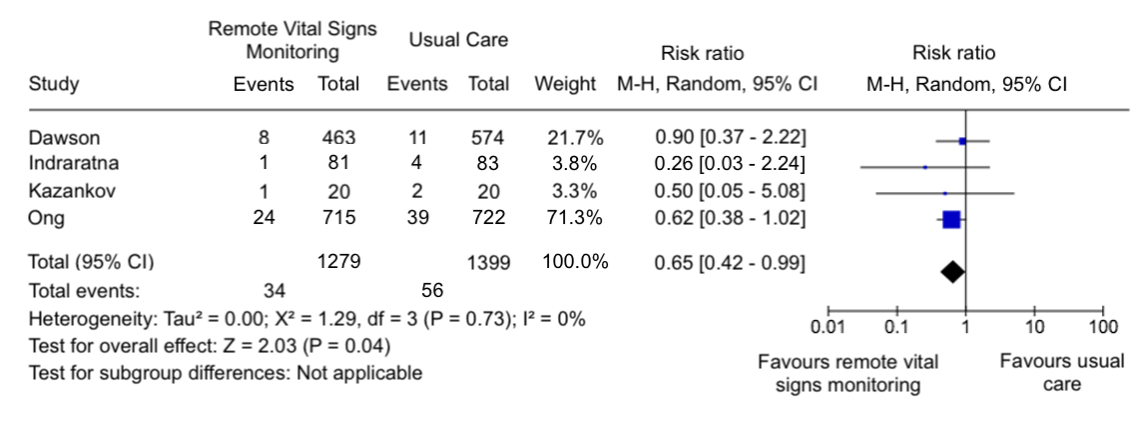
Forest plot of 4 [25,26,29,35] studies comparing remote vital signs monitoring against usual care on 30 day or less mortality.

Conversely, across 4 studies [25,26,29,35] reporting mortality within the initial 30 days, there was a significant reduction in mortality rates (RR 0.65, 95% CI 0.42-0.99; Figure 3). There was no significant heterogeneity noted in mortality within the first 30 days (*I*^2^=0%). However, publication bias for mortality was noted in the funnel plot (E Figure S2 in Multimedia Appendix 2). Certainty of evidence (GRADE) is very low (E Table S6 in Multimedia Appendix 2).

### Usability

Usability results were provided in 7 (25%) studies [26,27,33-35,42,43]. Ease of use was reported in 3 (11%) studies [26,27,35], patient satisfaction in 4 (14%) studies [26,33,34,43], and usefulness in 3 (11%) studies [27,35,42]. Usability results were obtained using questionnaires in 3 (11%) studies [27,35,36], surveys (method not described) in 2 (7%) studies [42,43], and phone surveys in 2 (7%) studies [27,33]. Most patients demonstrated compliance with home VSM and reported finding it easy to use. In addition, they expressed high levels of satisfaction and perceived the service as useful.

### HAH-Specific Studies

Only 2 studies [40,51], which were both case series, were conducted in the HAH setting. Both were abstract and involved patients with hematological malignancies.

Pauldo et al [51], described 20 patients readmitted from a HAH program, remote VSM (heart rate, blood pressure, weight, oxygen saturation, and temperature), and neurologic symptoms following chimeric antigen receptor T-cell therapy. The frequency of VSM or doctor, or nursing visits was not reported. Of the 20 patients, 13 (65%) had VSM alerts 48 hours before RTH. For the remaining 7 (35%) patients, 3 (15%) were RTH within 24 hours without significant VSM data. The other 4 (20%) were RTH for the following reasons: acute pancreatitis (n=1, 5%), acute pain crisis (n=1, 5%), a doubling of C-reactive protein (n=1, 5%), and neutropenic fever (n=1, 5%; the patient did not report VSM data).

Routledge et al [40], described 54 patients admitted to a HAH program with daily doctor visits and twice-daily nursing visits. Remote VSM included temperature, heart rate, blood pressure, and oxygen saturation. The frequency of VSM was not reported. A total of 47 (94%) patients were RTH, with 1 (2%) requiring intensive care support. No deaths were reported. The top reasons for RTH were fever (80%) and diarrhea (81%).

## Discussions

### Principal Findings

This systematic review and meta-analysis suggest that remote VSM in postacute patients or those in HAH programs is not significantly associated with a reduction in hospital readmission but may be linked to a reduction in mortality. However, these findings are limited by significant heterogeneity and varying study quality with a very low grade of certainty.

While VSM has been extensively studied and validated in the inpatient setting [2], its effectiveness in postacute populations or within HAH programs remains uncertain [52]. A key finding from our review is a lack of high-grade studies in this field. Out of the 28 identified published studies [24-51], only 12 [25-29,32,34,35,39,43,45,50] were available in full-text publications, with the remaining 16 [24,30,31,33,36-38,40-42, 44,46-49,51] being abstracts that were not subsequently published. Among the 12 full-text publications [25-29,32,34, 35,39,43,45,50], only 5 [25-29] were RCTs.

This review builds on the findings of a recent systematic review by Patel et al [53], describing and comparing remote VSM used in admission avoidance HAH trials. Patel et al [53] categorized studies investigating the effect of HAH versus inpatient stay based on the VSM approach used (unspecified, continuous, or intermittent) and made indirect comparisons of the effects of these 3 VSM approaches on mortality and RTH. Our study adds to this understanding of response to abnormal vitals, usability measures, and further highlights gaps in reporting and evidence base in remote vital signs monitoring in caring for acutely ill patients at home.

### Effect of Remote VSM on Clinical Outcomes

Our results indicate a limited benefit of remote VSM in terms of reducing readmissions within 60 days. This may be because, in the majority of cases, symptoms manifest before substantial changes in vital signs emerge. As such, symptomatic patients are likely to seek medical attention before abnormalities in vital signs are detected. Even in hospital settings, alterations in vital signs frequently remain undetected until reaching critical stages [54,55]. Although publication bias was noted, it is unlikely that unpublished studies would have exaggerated the effect of remote VSM on readmissions, considering the overall negative findings.

Conversely, remote VSM showed a statistically significant reduction in mortality within 30 days. This may be due to remote VSM serving as a safety net for patients who might have overlooked their symptoms or delayed seeking medical attention. However, only 4 [25,26,29,35] studies investigated mortality within 30 days or earlier, and the small sample sizes in studies by Indraratna et al [26] and Kazankov et al [35] may have an outsized influence on the overall result when included in the meta-analysis. The 2 larger studies (Dawson et al [25] and Ong et al [29]) did not show a statistically significant difference individually. Publication bias toward overestimating the effect of remote VSM on mortality was also observed (E Figure S2 in Multimedia Appendix 2), which would have exaggerated the perceived benefit of remote VSM on mortality. However, the power of the funnel plot asymmetry test is questionable, given that only 4 studies were included in the analysis.

### Challenges and Complexities in Clinical Application of Remote VSM

Although VSM is likely to play an increasingly important role in HAH and postacute care, this review further highlights several challenges in evaluating its clinical utility. VSM is a multifaceted intervention. While changes in vital signs signal potential clinical deterioration, the response to this signal determines the outcome and thus utility of remote VSM [56]. The quality of these signals, especially in the home setting, is of critical importance. The accuracy and reliability of continuous VSM technology, such as wearables (eg, Apple Watch) [57] are disputed, while the accuracy of patient-performed intermittent VSM in both recording and reporting remains questionable.

Critical illness scores like NEWS2 (National Early Warning Score 2) score, APACHE (Acute Physiology and Chronic Health Evaluation), and SOFA (Sequential Organ Failure Assessment) have been validated for predicting deterioration in hospital settings [58], but whether these thresholds apply to the ambulatory setting, where patients are more mobile and may experience greater physiological variation, is unclear [59]. The optimal frequency of remote VSM is also uncertain. There is limited literature comparing continuous readings via wearables to less frequent monitoring (1-4 times a day). Furthermore, the frequency and ease of remote VSM also affect patient adherence to VSM. Frequent VSM or difficult-to-use VSM modalities may result in noncompliance, undermining the effectiveness of the system. Even with clear thresholds of intervention and the most accurate and reliable VSM modalities, a lack of patient compliance could render remote VSM ineffective in improving patient safety. These aspects were not adequately addressed in the studies included in this review, but are critical to the pathway from signal to action to outcome.

### Limitations

This review has several limitations. First, a major limitation is the significant heterogeneity in study design, the types of vital signs monitored, and response to abnormal vital signs across studies. Although all studies measured some form of vital signs, there was no consistent set of parameters measured across studies. Moreover, interventions in response to abnormal vitals were poorly described in most studies. When interventions were mentioned, they ranged from physical review to alerting the patient’s general practitioner. This significant variation likely influenced the outcomes related to mortality and readmission. For example, common infections like urinary tract infections may only present with tachycardia in its early, easily treatable stages [59], which could be missed if the study focused solely on blood pressure. Alternatively, tachycardia and narrowed pulse pressure are the first signs of hypovolemic shock [60]. If this vital sign trigger was noted and intervened early, a patient in the early stages of hypovolemic shock might have been resuscitated early, with the prevention of readmission or mortality. Second, only 2 case series directly investigated VSM in the HAH setting. As a result, the literature search was expanded to include studies on patients in the postacute phase. However, this led to the majority of studies focusing on patients in the postacute phase rather than HAH specifically. Consequently, the results of this review may have limited applicability to the HAH setting. Finally, many included studies were abstracts. We hypothesize that remote VSM is a relatively new field of study, and many studies may still be in the conference presentation stage, explaining the prevalence of abstracts. These abstracts were included to ensure that potential prepublication data were not overlooked. However, abstracts often lack detailed methodology and results, which limits the quality of the study and the reliability of the data included.

### Future Research Directions

Future research on remote VSM should focus on the HAH setting including direct comparisons of all remote VSM (blood pressure, heart rate, oxygen saturation, and temperature) with consistent monitoring frequency and clear intervention protocols versus no VSM in HAH. Potential areas of research include comparisons between different monitoring modalities, such as self-monitoring, wearable devices, and provider-initiated monitoring, or looking at the accuracy and clinical validity of popular remote VSM tools, for example, smartwatches, to ensure their reliability and effectiveness in the HAH setting. Technological advancements, such as 5G networks and artificial intelligence, could offer new opportunities in this space. In addition, studies should determine the optimal frequency of remote VSM in the HAH setting that balances patient safety with ease of use while establishing clear and evidence-based thresholds for escalating care when specific vital sign parameters are reached.

### Conclusion

Remote VSM for patients in the postacute phase of illness did not result in a reduction of the 60-day readmission rate, although it was associated with a slight decrease in mortality within 30 days or less. The available published data on the impact of remote VSM in acutely ill patients at home are limited. Future studies are indicated to better understand how to integrate remote VSM into HAH programs.

## Appendix

Multimedia Appendix 1 PRISMA checklist.

Multimedia Appendix 2 Literature search strategy and supplementary tables and figures.

## Abbreviations

APACHE: Acute Physiology and Chronic Health Evaluation
GRADE: Grading Recommendations Assessment, Development, and Evaluation
HAH: hospital at home
NEWS2: National Early Warning Score 2
PRISMA: Preferred Reporting Items for Systematic Reviews and Meta-Analyses
PROSPERO: International Prospective Register of Systematic Reviews
RCT: randomized controlled trial
ROB: risk of bias
RR: risk ratio
RTH: return to hospital
SOFA: Sequential Organ Failure Assessment
VSM: vital signs monitoring
