# Supplementary material for: The Value of Remote Vital Signs Monitoring in Detecting Clinical Deterioration in Patients in Hospital at Home Programs or Postacute Medical Patients in the Community: Systematic Review
Source: J Med Internet Res. 2025 May 26;27:e64753. doi: 10.2196/64753 (PMC12149774; doi:10.2196/64753)
Supplement: Multimedia Appendix 2 [file jmir_v27i1e64753_app2.docx]

**Multimedia Appendix 2**

**Supplemental Methods 1 Literature Search Strategy 2**

**Supplemental Tables**

**E Table S1 4**

**E Table S2 11**

**E Table S3 17**

**E Table S4 21**

**E Table S5 24**

**E Table S6 27**

**E Table S7 28**

**E Table S8 29**

**Supplemental Figures**

**E Figure S1 30**

**E Figure S2 30**

**References 31**

**Supplemental Methods**

In this supplement, we describe the search strategy that was used as well as additional results from the systematic review.

The literature search strategy used for Embase, Pubmed and Scopus are described.

E table S1 describes the baseline characteristics of the included studies.

E table S2 describes the outcome, results and quality of studies.

E table S3 describes the device used, transmission type and parameters measured of the various studies.

E Table S4 reports on the risk of bias analysis of the randomised controlled trials included in the systematic review.

E Table S5 reports on the quality of cohort studies included in the systematic review.

E Table S6 reports on the quality of case series included in the systematic review.

E Table S7 reports on the GRADE framework for describing the certainty of evidence and justifying downgrading or upgrading of remote vital signs monitoring on 60 day readmission.

E Table S8 reports on the GRADE framework for describing the certainty of evidence and justifying downgrading or upgrading of remote vital signs monitoring on 30 day mortality.

E Figure S1 shows the funnel plot looking at publication bias for hospital readmission between 30 to 60 days.

E Figure S2 shows the funnel plot looking at publication bias for mortality within 30 days.

The completed PRISMA checklist is reported.

**Literature search strategy**

Embase: (‘hospital discharge'/exp OR 'transitional care'/exp OR 'postadmission':ab,ti,kw OR 'post-admission':ab,ti,kw OR 'post admission':ab,ti,kw OR 'after admission':ab,ti,kw OR 'after discharge':ab,ti,kw OR 'post discharge':ab,ti,kw OR 'post-discharge':ab,ti,kw OR 'postdischarge':ab,ti,kw OR 'after hospital*':ab,ti,kw OR 'posthospital*':ab,ti,kw OR 'post-hospital*':ab,ti,kw OR 'post hospital*':ab,ti,kw OR 'postacute':ab,ti,kw OR 'post-acute':ab,ti,kw OR 'post acute':ab,ti,kw OR 'Transition care':ab,ti,kw OR ('acute*':ab,ti,kw AND 'home':ab,ti,kw AND 'care':ab,ti,kw AND 'hospital':ab,ti,kw) OR 'hospital at home':ab,ti,kw OR 'hospital in the home':ab,ti,kw OR 'hospital-at-home':ab,ti,kw OR 'home hospital*':ab,ti,kw OR 'hospital in home':ab,ti,kw OR 'hospital-in-home':ab,ti,kw OR 'hospital-in-the-home':ab,ti,kw OR 'early supported discharge':ab,ti,kw OR 'admission avoidance':ab,ti,kw) AND ('ambulatory monitoring'/exp OR 'monitor*':ab,ti,kw OR 'measur*':ab,ti,kw) AND ('vital sign'/exp OR 'vital sign*':ab,ti,kw OR 'blood pressure*':ab,ti,kw OR 'heart rate*':ab,ti,kw OR 'temperature':ab,ti,kw OR 'oxygen saturation':ab,ti,kw OR 'respiratory rate':ab,ti,kw OR 'pulse rate*':ab,ti,kw) AND (2012:py OR 2014:py OR 2015:py OR 2016:py OR 2017:py OR 2018:py OR 2019:py OR 2020:py OR 2021:py OR 2022:py) AND [english]/lim

Pubmed: (Home Care Services, Hospital-Based[MeSH Terms] OR "Patient Discharge"[Mesh] OR (patient*[Title/Abstract] AND discharg*[Title/Abstract] AND hospital*[Title/Abstract]) OR postadmission[Title/Abstract] OR post-admission[Title/Abstract] OR post admission [Title/Abstract] OR after admission[Title/Abstract] OR after discharge[Title/Abstract] OR post discharge[Title/Abstract] OR post-discharge[Title/Abstract] OR postdischarge[Title/Abstract] OR after hospital*[Title/Abstract] OR posthospital*[Title/Abstract] OR post-hospital*[Title/Abstract] OR post hospital*[Title/Abstract] OR postacute[Title/Abstract] OR post-acute[Title/Abstract] OR post acute[Title/Abstract] OR "Transitional Care"[Mesh] OR Transition care[Title/Abstract] OR (acute*[Title/Abstract] AND home[Title/Abstract] AND care[Title/Abstract] AND hospital[Title/Abstract]) OR hospital at home[Title/Abstract] OR hospital in the home[Title/Abstract] OR hospital-at-home[Title/Abstract] OR home hospital*[Title/Abstract] OR hospital in home[Title/Abstract] OR hospital-in-home[Title/Abstract] OR hospital-in-the-home[Title/Abstract] OR early supported discharge[Title/Abstract] OR admission avoidance[Title/Abstract]) AND ("Monitoring, Ambulatory"[Mesh] OR "Telemetry"[Mesh] OR “oximetry" [MeSH] OR "Telemetry"[Mesh] OR "oximetry" [MeSH] OR (telemetry[Title/Abstract] OR oximetry[Title/Abstract] OR monitor*[Title/Abstract]) OR measur*[Title/Abstract]) AND ("Vital Signs"[Mesh] OR vital sign*[Title/Abstract] OR blood pressure*[Title/Abstract] OR heart rate*[Title/Abstract] OR temperature[Title/Abstract] OR oxygen saturation[Title/Abstract] OR respiratory rate[Title/Abstract] OR pulse rate*[Title/Abstract])

Scopus: (TITLE-ABS-KEY(hospital AND patient AND discharge) OR TITLE-ABS-KEY(“transitional care”) OR TITLE-ABS-KEY(“hospital at home”) OR TITLE-ABS-KEY(“hospital in the home”) OR TITLE-ABS-KEY(“hospital-at-home”) OR TITLE-ABS-KEY(“home hospital*”) OR TITLE-ABS-KEY(“hospital in home”) OR TITLE-ABS-KEY(“hospital-in-home”) OR TITLE-ABS-KEY(“hospital-in-the-home”) OR TITLE-ABS-KEY(“early supported discharge”) OR TITLE-ABS-KEY(“admission avoidance”)) AND (TITLE-ABS-KEY(“monitor*”) OR TITLE-ABS-KEY(“measur*”)) AND (TITLE-ABS-KEY(“vital sign”) OR TITLE-ABS-KEY(“blood pressure*”) OR TITLE-ABS-KEY(“heart rate*”) OR TITLE-ABS-KEY(“temperature”) OR TITLE-ABS-KEY(“oxygen saturation”) OR TITLE-ABS-KEY(“respiratory rate”) OR TITLE-ABS-KEY(“pulse rate*”)) AND PUBYEAR > 2012 AND ( LIMIT-TO ( LANGUAGE , "English" ) )

**E Table S1.** Baseline description of studies.

| Author | Year | Study Design | | Study setting | Condition studied | Intervention | Control | Intervention if abnormal vitals |
| --- | --- | --- | --- | --- | --- | --- | --- | --- |
| Kimchi A et al [24] | 2019 | Randomised Controlled Trial | Abstract | Postacute | Heart failure | No information (NI) | Usual Care | NI |
| Dawson NL et al [25] | 2021 | Randomised Controlled Trial | Full text | Postacute | Heart failure | Daily vitals sign monitoring (VSM) via home installed vitals equipment | Usual care | No intervention from investigation team |
| Indraratna P et al [26] | 2002 | Randomised Controlled Trial | Full text | Postacute | 1. Acute coronary syndrome 2. Heart failure | Daily VSM via smartphone application with Bluetooth enabled devices | Usual care | Yellow alert: Contact depending on discretion Red alert: Contact and decide if to inform General Practioner (GP)  /Cardiologist |
| Noel K et al [27] | 2020 | Randomised Controlled Trial | Full text | Postacute | Multiple co-morbid diseases | 1. Daily VSM via smartphone application with Bluetooth enabled devices 2. Weekly video consult | Usual Care | Refer GP |
| Olivari Z GS et al [28] | 2014 | Randomised Controlled Trial | Full text | Postacute | Heart failure | NI | Usual Care | NI |
| Ong MK et al [29] | 2016 | Randomised Controlled Trial | Full text | Postacute | Heart failure | Daily VSM via wireless transmission device | Usual Care | Review by medical team with referral to GP or emergency department (ED) if needed |
| Rosu R D et al [30] | 2020 | Randomised Controlled Trial | Abstract | Postacute | Heart failure | NI | Usual Care | NI |
| Schulte P et al [31] | 2020 | Randomised Controlled Trial | Abstract | Postacute | Heart failure | Daily VSM via wireless transmission device | Usual Care | NI |
| A Nunes Ferreria et al [32] | 2019 | Nested cohort | Full text | Postacute | Heart failure | 1. Daily VSM via smartphone application with Bluetooth enabled devices 2. Daily phone consult | Usual Care | NI |
| Brennan K et al [33] | 2021 | Prospective cohort | Abstract | Postacute | Heart failure | 1. Daily VSM via smartphone application with Bluetooth enabled devices 2. Daily tele-consults | Usual Care | NI |
| Broad J et al [34] | 2014 | Propensity match | Full text | Postacute | Heart failure | Daily VSM with tele-monitoring | Usual Care | Primary physician updated |
| Kazankov K et al [35] | 2023 | Prospective cohort | Full text | Postacute | Acute liver decompensation | 1. Daily VSM via smartphone application with Bluetooth enabled devices 2. Daily video consult | Usual Care | NI |
| McClinton K et al [36] | 2015 | Prospective cohort | Abstract | Postacute | Chronic co-morbidities | 1. Weekly VSM 2. Weekly phone consult | Usual Care | NI |
| Mora EV et al [37] | 2015 | Prospective cohort | Abstract | Postacute | Heart failure | Daily VSM with tele-monitoring | Usual Care | NI |
| Prabhakar et al [38] | 2020 | Propensity match | Abstract | Postacute | Heart failure | Daily VSM via tele-monitoring (not specified) | Usual Care | NI |
| Bennett MK et al [39] | 2017 | Case series | Full text | Postacute | Heart failure | Contactless under-the-mattress piezoelectric sensor to monitor physiological vibrations | Usual Care | NI |
| Routledge D et al [40] | 2021 | Case series | Abstract | Hospital at Home | Autologous stem cell transplant | 1. Daily physician review 2. Twice daily nursing review | Usual Care | Nursing physical review |
| Power D et al [41] | 2014 | Case series | Abstract | Postacute | NI | Health care team visits up to 3 times a day with monitoring equipment transmitting vitals directly to the cloud via 3G technology | Usual Care | Nursing phone review |
| Berry E [42] | 2017 | Case series | Abstract | Postacute | Heart failure | Daily VSM via Bluetooth enabled devices with wireless cutaneous telemetry sensor | Usual Care | Physician phone review |
| Ho K et al [43] | 2021 | Case series | Full text | Postacute | Heart failure | Self-entry of daily VSM | Usual Care | 1.Nursing phone review 2. Discussion with physician regarding medication changes |
| Mitchell JS et al [44] | 2014 | Case series | Abstract | Postacute | Heart failure | Daily self-reported VSM | Usual Care | NI |
| Kargiannakis M et al [45] | 2017 | Case series | Full text | Postacute | Chronic obstructive pulmonary disease | Daily VSM via smartphone application with Bluetooth enabled devices | Usual Care | NI |
| Hsu MS [46] | 2014 | Case series | Abstract | Postacute | Chronic co-morbidities | Daily self-reported VSM | Usual Care | Nursing or physician phone review |
| Dickinson M et al [47] | 2015 | Case series | Abstract | Postacute | Heart failure | 1. Daily VSM via Cardiocom HT devices 2. Daily heart failure disease management questions | Usual Care | Nursing phone review |
| Proctor S et al [48] | 2021 | Case series | Abstract | Postacute | Chronic obstructive pulmonary disease | Under-mattress sleep monitoring (EarlySense) | Usual Care | NI |
| Sandbaek A et al [49] | 2022 | Case series | Abstract | Postacute | Pneumonia | Day 2, day 4 and day 10 respiratory checks by nurses | Usual Care | NI |
| Stehlik J et al [50] | 2020 | Case series | Full text | Postacute | Heart failure | Disposable multisensor patch over chest which uploads continuous data | Usual Care | NI |
| Paludo J et al [51] | 2021 | Case series | Abstract | Hospital at  home | Chimeric antigen receptor T-cell therapy | 1. Continuous VSM via in home, electronic health record-integrated technology. 2. Neurologic symptom reporting | Usual Care | NI |

NI: No information

VSM: vital signs monitoring

GP: general practitioner

ED: Emergency department

**E Table S2.** Description of outcome, results and quality of studies

| Study | Clinical Outcomes | Intervention | Control |  |  | Usability | | | Quality of study |
| --- | --- | --- | --- | --- | --- | --- | --- | --- | --- |
|  |  | Events, n | Events, n | Relative Risk (95% CI) | *P* | Outcomes | Result | Method |  |
| Indraratna P et al[26] | 30 day hospital readmission | 11 (81) | 11 (83) | 1.02 (0.46-2.24) | 0.96 | Ease of use | 64/67 found the app easy or very easy to use | Survey | Some risk of bias |
|  | 6 month hospital readmission | 21 (81) | 41 (83) | 0.52 (0.34- 0.80) | 0.003 | Satisfaction | 4.56/5 stars |  |  |
|  | 30 day mortality | 1 (81) | 4 (83) | 0.25 (0.03- 2.24) | 0.23 |  |  |  |  |
| Dawson NL et al[25] | 30 day hospital readmission | 81 (476) | 129 (574) | 0.75 (0.58- 0.97) | 0.03 | No information (NI^c^) | NI | NI | High risk of bias |
|  | 30 day mortality | 8 (463) | 11 (574) | 0.90 (0.36 - 2.22) | 0.82 |  |  |  |  |
| Noel K et al[27] | 30 days hospital readmission | NI (45) | NI (57) | 2.66 (0.41 - 17.3) | 0.31 | Perception of difficulty | No perception of difficulty p > 0.072 | Phone Survey | Some risk of bias |
|  |  |  |  |  |  | Enthusiasm and confidence in tele health | Improvement in enthusiasm and confidence in tele health p = 0.0001 |  |  |
| Ong MK et al[29] | 30 day hospital readmission | 162 (715) | 156 (722) | 1.04 (0.87 - 1.27) | 0.631 | NI | NI | NI | Some risk of bias |
|  | 6 month hospital readmission | 363 (715) | 355 (722) | 1.03 (0.93 - 1.14) | 0.54 |  |  |  |  |
|  | 30 day mortality | 24 (715) | 39 (722) | 0.62 (0.38 - 1.02) | 0.06 |  |  |  |  |
| Olivari Z GS et a^l^[28] | 12 month readmission for heart failure (HF^b^) | 79 (229) | 43 (110) | 0.88 (0.66 - 1.18) | 0.097 | NI | NI | NI | Some risk of bias |
|  | 12 month mortality | 55 (229) | 24 (110) | 1.1 (0.72 - 168) | 0.48 |  |  |  |  |
| Schulte P et al[31] | 60 day hospital readmission or mortality | 26% | 28% | No effect | 0.77 | NI | NI | NI | High risk of bias |
|  | 60 day mortality | 2% | 7% | No effect | 0.2 |  |  |  |  |
| Rosu R D et al[30] | 45 day hospital readmission | 4 (20) | 2 (25) | 2.5 (0.51 - 12.2) | 0.26 | NI | NI | NI | High risk of bias |
| Kimchi A et al [24] | 30 day hospital readmission | NI | NI | No effect | NI | NI | NI | NI | High risk of bias |
|  | 6 month hospital readmission | NI | NI | No effect | NI |  |  |  |  |
|  | 6 month mortality | NI | NI | No effect | NI |  |  |  |  |
| Kazankov et al[35] | 10 day hospital readmission | 8 (20) | 13 (20) | 0.36 (0.10 - 1.29) | 0.12 | Ease of use | Median score 9/10 | Survey | Good quality |
|  | 10 day mortality | 1 (20) | 2 (20) | 0.473 (0.04 - 5.68) | 0.59 |  |  |  |  |
| Broad J et al[34] | 30 days hospital readmission (HF) | 5 (59) | 10 (59) | 0.45 (0.15 - 1.42) | 0.09 | Ease of use | 4.98/5 (HF) 4.99/5 (COPD^a^) | Survey | Good quality |
|  | 90 day hospital readmission (HF) | 15 (59) | 19 (59) | 0.72 (0.32 - 1.60) | 0.12 |  |  |  |  |
|  | 6 month hospital readmission (HF) | 21 (59) | 26 (59) | 0.7 (0.34 - 1.47) | 0.1 |  |  |  |  |
|  | 30 day hospital readmission Chronic Obstructive Pulmonary Disease (COPD) | 6 (58) | 38 (174) | 0.41 (0.17 - 1.04) | 0.02 |  |  |  |  |
|  | 90 day hospital readmission (COPD) | 21 (58) | 69 (174) | 0.86 (0.47 - 1.60) | 0.11 |  |  |  |  |
|  | 6 month hospital readmission (COPD) | 27 (58) | 93 (174) | 0.76 (0.42 - 1.38) | 0.08 |  |  |  |  |
| Nunes Ferreria et al[32] | 12 month hospital readmission or mortality | 5 (25) | 28 (50) | 0.27 (0.11 - 0.71) | <0.01 | NI | NI | NI | Good quality |
| Prabhakar et al[38] | 30 days hospital readmission | NI | Ni | No significant difference | NI | NI | NI | NI | Good quality |
| Mora EV et al[37] | Readmission or hospital presentation | NI | NI | 0.41 (0.17 - 0.99) | NI | NI | NI |  | Poor quality |
| McClinton et al [36] | 30 day hospital readmission | 23 (270) | 1441 (5148) | 0.24 (0.16 - 0.37) | < 0.01 | NI | NI | NI | Good quality |
| Brennan et al [33] | 30 day hospital readmission | NI | NI | 24.6% reduction | NI | Satisfaction | 97.5% reported satisfied with the program | Phone survey | Fair quality |
| Stehlik et al [50] | 90 day hospital readmission | 38 (100) | NI | NI | NI | NI | NI | NI | Score of 5 |
| Kargiannakis et al [45] | 28 day hospital readmission | 2 (23) | NI | NI | NI | NI | NI | NI | Score of 5 |
| Bennett et al [39] | 30 day hospital readmission | 9 (29) | NI | NI | NI | Tolerance of home monitoring | 97% | NI | Score of 5 |
| Ho et al [43] | 90 day hospital readmission | 87% decrease | NI | NI | NI | Satisfaction | Mean score 80, median 81.4 | Survey | Score of 5 |
| Sandbaek A et al [49] | 10 day hospital readmission | 16 (50) | NI | NI | NI | NI | NI | NI | Score of 3 |
| Routledge et al [40] | Bed days saved per patient | 9 beds day saved | NI | NI | NI | NI | NI | NI | Score of 4 |
| Paludo et al [51] | Hospital readmission | 20 | NI | NI | NI | NI | NI | NI | Score of 1 |
| Proctor et al [48] | 30 day hospital readmission | 7 (26) | NI | NI | NI | NI | NI | NI | Score of 4 |
|  | 90 day hospital readmission | 17 (26) | NI | NI | NI |  |  |  |  |
| Dickinson et al [47] | 50 day hospital readmission | 50/138 | NI | NI | NI | NI | NI | NI | Score of 3 |
|  | 170 day hospital readmission | 91/138 | NI | NI | NI |  |  |  |  |
| Power D et al [41] | 52 day hospital readmission | 2 (13) | NI | NI | NI | NI | NI | NI | Score of 4 |
| Mitchell et al [44] | 30 day hospital readmission | 13% | NI | NI | NI | NI | NI | NI | Score of 3 |
|  | 30 day mortality | 6% | NI | NI | NI |  |  |  |  |
| Hsu et al [46] | Length of stay | LOS reduced by 21% | NI | NI | NI | NI | NI | NI | Score of 3 |
| Berry et al [42] | 30 day hospital readmission | 5 (24) | NI | NI | NI | Helpfulness | 24/31 found it helpful | Survey | Score of 4 |

Foot note

^a^COPD: chronic obstructive pulmonary disease

^b^HF: heart failure

^c^NI: no information

**E Table S3**: Vital sign monitoring device, transmission type and parameters measured of included studies

NI: No information

|  | Device | | | Transmission Type | | Parameters Measured | | | | | | |
| --- | --- | --- | --- | --- | --- | --- | --- | --- | --- | --- | --- | --- |
| Author | Smartphone app | Phone call | Video Call | Bluetooth | Self-entry | BP | HR | Weight | Pulse Oximetry | Blood glucose | ECG | Others |
| Indraratna et al [26] | x |  |  | x |  | x | x | x |  |  |  |  |
| Dawson et al [25] | x |  |  |  |  | x | x | x | x | x |  |  |
| Noel et al [27] | x |  | x | x |  | x | x | x | x |  |  |  |
| Ong et al [29] | x |  |  | x |  | x | x | x |  |  |  |  |
| Schulte et al [31] | x |  |  | x |  | x | x | x |  |  |  | Respiratory rate |
| Rosu et al [30] | x |  |  | x |  | x | x | x |  |  | x |  |
| Kimchi et al [24] | x |  |  | x |  | NI |  |  |  |  |  |  |
| Olivari ZGS et al [28] | x |  |  | x |  | x | x | x | x |  | x |  |
| Kazankov et al [35] | x | x | x | x | x | x | x | x |  |  | x | Bioimpedance |
|  |  |  |  |  |  |  |  |  |  |  |  | Hepatic encephalopathy assessment |
|  |  |  |  |  |  |  |  |  |  |  |  | Well being |
|  |  |  |  |  |  |  |  |  |  |  |  | Food, fluid and alcohol intake |
| Broad et al [34] | NI |  |  | NI |  |  |  | x |  |  |  | Symptoms |
| Prabhakar et al [38] | x | x |  | x | x | x |  | x |  |  |  |  |
| Nunes Ferreria et al [32] | x |  |  | x |  | x | x |  |  |  |  |  |
| Mora EV et al [37] | NI |  |  | NI |  |  |  |  | x |  |  | Gait speed |
| McClinton et al [36] |  | x |  |  | x | x | x | x |  |  |  |  |
| Brennan et al [33] | x | x |  | x |  | x | x |  | x |  |  | Temperature |
| Stehlik et al [50] | x |  |  | x |  |  | x |  | x |  | x | Temperature, |
|  |  |  |  |  |  |  |  |  |  |  |  | Gross activity |
|  |  |  |  |  |  |  |  |  |  |  |  | Walking |
|  |  |  |  |  |  |  |  |  |  |  |  | Sleep |
|  |  |  |  |  |  |  |  |  |  |  |  | Body tilt and posture |
|  |  |  |  |  |  |  |  |  |  |  |  | Respiratory rate |
| Kargiannakis et al [45] | x |  |  | x | x | x | x | x | x |  |  | HF symptoms |
| Sandbaek A et al [49] | x |  |  | NI |  |  |  |  |  |  |  | Respiratory rate |
| Routledge et al [40] |  |  |  |  |  | x | x |  | x |  |  | Temperature |
| Paludo, 2021 [51] | x |  |  | x |  | x | x | x | x |  |  | New neurological symptoms |
| Proctor, 2021 [48] | Under-mattress sleep monitoring (EarlySense) |  |  | NI |  |  | x |  |  |  |  | Stage of sleep |
|  |  |  |  |  |  |  |  |  |  |  |  | Respiratory rate |
| Ho et al [43] | Vitals keyed into touchscreen tablet |  |  |  | x | x |  | x | x |  |  | HF symptoms |
| Dickinson et al [47] | x |  |  | x |  | x | x | x | x |  |  | HF symptoms |
| Power D et al [41] | x |  |  | x |  | x | x | x | x |  |  |  |
| Mitchell et al [44] | x | x |  |  | x | x | x | x |  |  |  | HF symptoms |
| Hsu et al [46] |  | x |  |  | x |  |  |  | NI |  |  | Symptoms |
| Berry et al [42] | x |  |  | x | x | x |  |  | x |  | x | Symptoms |
| Bennett et al [39] | x |  |  | x |  |  | x |  |  |  |  | Behaviour score |
|  |  |  |  |  |  |  |  |  |  |  |  | Movement rate |
|  |  |  |  |  |  |  |  |  |  |  |  | Rapid and shallow respiratory rate |

**E Table S4**: risk of bias analysis of the randomised controlled trials included in the systematic review

Y: Yes, N: No, NI: No information, PY: Probably yes, PN: Probably no

|  |  | Indraratna P et al [26] | Dawson NL et al [25] | Noel K et al [27] | Ong MK et al [29] | Olivari Z GS et al [28] | Schulte P et al [31] | Rosu R D et al [30] | Kimchi A et al [24] |
| --- | --- | --- | --- | --- | --- | --- | --- | --- | --- |
| Section 1 | | Low risk of bias | Low risk of bias | Low risk of bias | Some concerns | Low risk of bias | Some concerns | Some concerns | Some concerns |
|  | 1.1 | Y | Y | Y | Y | Y | Y | Y | Y |
|  | 1.2 | Y | Y | Y | NI | Y | NI | NI | NI |
|  | 1.3 | N | N | N | N | N | NI | N | NI |
| Section 2 | | Low risk of bias | Low risk of bias | Low risk of bias | Low risk of bias | Low risk of bias | High risk of bias | High risk of bias | High risk of bias |
|  | 2.1 | Y | Y | Y | Y | Y | Y | Y | Y |
|  | 2.2 | Y | Y | Y | Y | Y | Y | Y | Y |
|  | 2.3 | N | N | N | N | N | N | N | NI |
|  | 2.4 | - | - | - | - | - | - | - | - |
|  | 2.5 | - | - | - | - | - | - | - | - |
|  | 2.6 | Y | Y | Y | Y | Y | N | NI | NI |
|  | 2.7 |  |  |  |  |  | Y | Y | NI |
| Section 3 | | Low risk of bias | High risk of bias | Low risk of bias | Low risk of bias | Low risk of bias | High risk of bias | Low risk of bias | Some risk of bias |
|  | 3.1 | Y | N | N | Y | Y | N | Y | NI |
|  | 3.2 | - | N | PY | - | - | N | - | NI |
|  | 3.3 | - | Y | - | - | - | Y | - | - |
|  | 3.4 | - | NI | - | - | - | Y | - | - |
| Section 4 | | Some risk of bias | Some risk of bias | Some risk of bias | Some risk of bias | Some risk of bias | Some risk of bias | Some risk of bias | Some risk of bias |
|  | 4.1 | N | N | N | N | N | N | N | N |
|  | 4.2 | N | N | N | N | N | N | N | N |
|  | 4.3 | PY | PY | PY | PY | PY | PY | PY | PY |
|  | 4.4 | Y | Y | Y | Y | Y | Y | Y | Y |
|  | 4.5 | PN | PN | PN | PN | PN | PN | PN | PN |
| Section 5 | | Low risk of bias | Low risk of bias | Low risk of bias | Low risk of bias | Low risk of bias | Low risk of bias | Low risk of bias | Low risk of bias |
|  | 5.1 | Y | Y | Y | Y | Y | Y | Y | PY |
|  | 5.2 | N | N | N | N | N | N | N | N |
|  | 5.3 | N | N | N | N | N | N | N | N |
|  | Overall | Some risk of bias | High risk of bias | Some risk of bias | Some risk of bias | Some risk of bias | High risk of bias | High risk of bias | High risk of bias |

1.1: Was the allocation sequence random?

1.2: Was the allocation sequence concealed until participants were enrolled and assigned to interventions?

1.3: Did baseline differences between intervention groups suggest a problem with the randomization process?

2.1 Were participants aware of their assigned intervention during the trial?

2.2 Were carers and people delivering the interventions aware of participants' assigned intervention during the trial?

2.3 If Y/PY/NI to 2.1 or 2.2: Were there deviations from the intended intervention that arose because of the trial context?

2.4 If Y/PY to 2.3: Were these deviations likely to have affected the outcome?

2.5. If Y/PY/NI to 2.4: Were these deviations from intended intervention balanced between groups?

2.6 Was an appropriate analysis used to estimate the effect of assignment to intervention?

2.7 If N/PN/NI to 2.6: Was there potential for a substantial impact (on the result) of the failure to analyse participants in the group to which they were randomized?

3.1 Were data for this outcome available for all, or nearly all, participants randomized?

3.2 If N/PN/NI to 3.1: Is there evidence that the result was not biased by missing outcome data?

3.3 If N/PN to 3.2: Could missingness in the outcome depend on its true value?

3.4 If Y/PY/NI to 3.3: Is it likely that missingness in the outcome depended on its true value?

4.1 Was the method of measuring the outcome inappropriate?

4.2 Could measurement or ascertainment of the outcome have differed between intervention groups?

4.3 If N/PN/NI to 4.1 and 4.2: Were outcome assessors aware of the intervention received by study participants?

4.4 If Y/PY/NI to 4.3: Could assessment of the outcome have been influenced by knowledge of intervention received?

4.5 If Y/PY/NI to 4.4: Is it likely that assessment of the outcome was influenced by knowledge of intervention received?

5.1 Were the data that produced this restuls analysed in accordance with a pre-specified analysis plan that was finalised before unblinded outcome data were available for analysis?

5.2 Is the numerical result being assessed likely to have been seslected on the basis of the results from multiple eligible outcomes wihtin the outcome domain?

5.3 Is the numerical result being assessed likely to have been seslected on the basis of the results from multiple eligible analyses of the data?

**E Table S5**: Quality of cohort studies included in the systematic review

|  | | Kazankov et al [35] | Nunes Ferreria et al [32] | Broad et al [34] | Prabhakar et al [38] | Mora EV et al [37] | McClinton et al [36] | Brennan et al [33] |
| --- | --- | --- | --- | --- | --- | --- | --- | --- |
| **Selection** | |  |  |  |  |  |  |  |
|  | Representativeness of the exposed cohort | Truly representative | Truly representative | Somewhat representative | Truly representative | No description of derivation of cohort | Truly representative | Selected group |
|  | Selection of the non-exposed cohort | Drawn from same community as the exposed cohort | Drawn from same community as the exposed cohort | Drawn from same community as the exposed cohort | Drawn from same community as the exposed cohort | No description of derivation of cohort | Drawn from same community as the exposed cohort | No description |
|  | Ascertainment of exposure | Secure record | Secure record | Secure record | Secure record | Secure record | Secure record | Secure record |
|  | Demonstration that outcome of interest was not present at start of study | Yes | Yes | Yes | Yes | Yes | Yes | Yes |
|  | Number of stars (Selection) | 4 | 4 | 3 | 4 | 2 | 4 | 2 |
| **Comparability** | |  |  |  |  |  |  |  |
|  | Comparability of cohorts on the basis of the design or analysis controlled for confounders | Controls for age, sex, marital status, Controls for other factors | Controls for other factors | Controls for age, sex, marital status, Controls for other factors | Controls for age, sex, marital status, Controls for other factors | Cohorts are not comparable on basis of design or analysis controlled for confounders | Controls for other factors | Controls for age, sex, marital status |
|  | Number of stars (Comparability) | 2 | 1 | 2 | 2 | 0 | 1 | 1 |
| **Outcome** | |  |  |  |  |  |  |  |
|  | Assessment of outcome | Record linkage | Record linkage | Record linkage | Record linkage | Record linkage | Record linkage | Record linkage |
|  | Was follow-up long enough for outcomes to occur | Yes | Yes | Yes | Yes | Yes | Yes | Yes |
|  | Adequacy of follow-up of cohorts | Subjects lost to follow up unlikely to introduce bias - ≤20% lost or description of lost no different from those followed | No statement | No statement | Subjects lost to follow up unlikely to introduce bias - ≤20% lost or description of lost no different from those followed | No statement | Complete follow up - all subjects accounted for | No statement |
|  | Number of stars (Outcome) | 3 | 2 | 2 | 3 | 2 | 3 | 2 |
| Overall study quality | | Good quality | Good quality | Good quality | Good quality | Poor quality | Good quality | Fair quality |

**E Table S6**: Quality of case series included in the systematic review

Y: Yes, N: No, NI: No information

|  | | Stehlik et al [50] | Bennett et al [39] | Kargiannakis et al [45] | Ho et al [43] | Sandbaek et al [49] | Routledge et al [40] | Paludo et al [51] | Proctor et al [48] | Dickinson et al [47] | Power D et al [41] | Mitchell et al [44] | Hsu et al [46] | Berry et al [42] |
| --- | --- | --- | --- | --- | --- | --- | --- | --- | --- | --- | --- | --- | --- | --- |
| Selection | |  |  |  |  |  |  |  |  |  |  |  |  |  |
|  | 1 | Y | Y | Y | Y | N | Y | N | Y | N | N | N | N | Y |
| Ascertainment | |  |  |  |  |  |  |  |  |  |  |  |  |  |
|  | 2 | Y | Y | Y | Y | Y | Y | Y | Y | Y | Y | Y | Y | Y |
|  | 3 | Y | Y | Y | Y | Y | Y | N | Y | Y | Y | Y | Y | Y |
| Causality | |  |  |  |  |  |  |  |  |  |  |  |  |  |
|  | 4 | N | N | N | N | NI | NI | NI | NI | NI | NI | NI | NI | NI |
|  | 5 | N | N | N | N | NI | NI | NI | NI | NI | NI | NI | NI | NI |
|  | 6 | N | N | N | N | N | N | N | N | N | N | N | N | N |
|  | 7 | Y | Y | Y | Y | NI | NI | NI | Y | Y | Y | Y | Y | Y |
| Reporting | |  |  |  |  |  |  |  |  |  |  |  |  |  |
|  | 8 | Y | Y | Y | Y | Y | Y | N | N | N | Y | N | N | N |
| Total Score | | 5 | 5 | 5 | 5 | 3 | 4 | 1 | 4 | 3 | 4 | 3 | 3 | 4 |

1. Does the patient(s) represent(s) the whole experience of the investigator (centre) or is the selection method unclear to the extent that other patients with similar presentation may not have been reported?
2. Was the exposure adequately ascertained?
3. Was the outcome adequately ascertained?
4. Were other alternative causes that may explain the observation ruled out?
5. Was there a challenge/rechallenge phenomenon?
6. Was there a dose–response effect?
7. Was follow-up long enough for outcomes to occur?
8. Is the case(s) described with sufficient details to allow other investigators to replicate the research or to allow practitioners make inferences related to their own practice?

| Domains for assessing certainty of evidence by outcome | Results section | Reasons for lowering or increasing the certainty of evidence |
| --- | --- | --- |
| Initial level of certainty | Mix of 4 randomised studies and 2 non-randomised studies | Begin at high certainty (systematic review) |
| Risk of bias | 2 randomised studies with some risk of bias, 2 randomised studies with high risk of bias, 2 non-randomised studies of good quality | Remain at high certainty. Majority of studies (4 out of 6) were of acceptable quality. |
| Inconsistency | Significant heterogeneity noted with I^2^ = 58%). | Reduce a grade to moderate certainty due to the significant heterogeneity noted between studies |
| Indirectness | Studies evaluated patients in the post-acute phase rather than hospital at home | Reduce a grade to low certainty as the studies were not directly evaluating patients in the hospital at home setting |
| Imprecision | 2 studies had positive results with 4 studies having negative results. Two studies had large confidence intervals (0.47 – 2.23 and 0.51 – 12.29). | Reduce a grade to very low certainty as discrepancy between studies in terms of results with 2 studies having large confidence intervals. |
| Publication bias | Publication bias was noted | Remain at very low certainty. Though publication bias was noted, the result was a negative result and would not be affected by publication bias |

**E Table S7:** Framework for describing the certainty of evidence and justifying downgrading or upgrading of remote vital signs monitoring against usual care on 60 day readmission.

| Domains for assessing certainty of evidence by outcome | Results section | Reasons for lowering or increasing the certainty of evidence |
| --- | --- | --- |
| Initial level of certainty | Mix of 3 randomised studies and 1 non-randomised studies | Begin at high certainty (systematic review) |
| Risk of bias | 2 randomised studies with some risk of bias, 1 randomised studies with high risk of bias, 1 non-randomised studies of good quality | Remain at high certainty. Majority of studies (3 out of 4) were of acceptable quality. |
| Inconsistency | No significant heterogeneity noted with I^2^ = 0%). | Remain at high certainty. As there was no significant heterogeneity noted between studies |
| Indirectness | Studies evaluated patients in the post-acute phase rather than hospital at home | Reduce to moderate certainty. The studies were not directly evaluating patients in the hospital at home setting |
| Imprecision | All studies had large confidence intervals | Reduce to low certainty due to the imprecision noted in the results. |
| Publication bias | Publication bias was noted | Reduce to very low certainty due to publication bias noted. |

**E Table S8:** Framework for describing the certainty of evidence and justifying downgrading or upgrading of remote vital signs monitoring against usual care on 30-day or less mortality.

**E Figure S1:** Funnel plot looking at publication bias in studies comparing remote vital signs monitoring against usual care on 60 day readmission


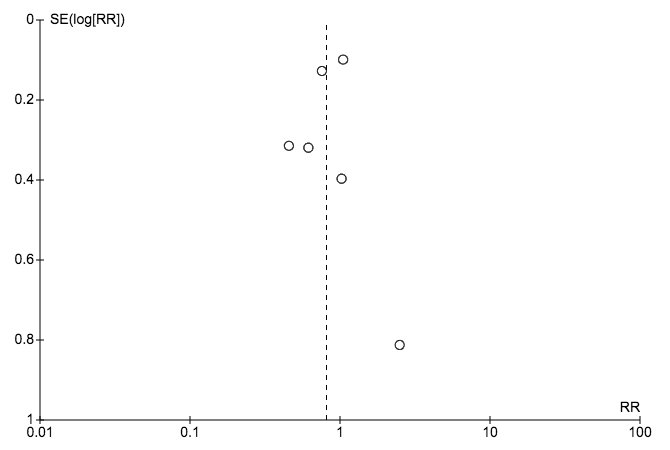


**E Figure S2:** Funnel plot looking at publication bias in studies comparing remote vital signs monitoring against usual care on 30 day or less mortality


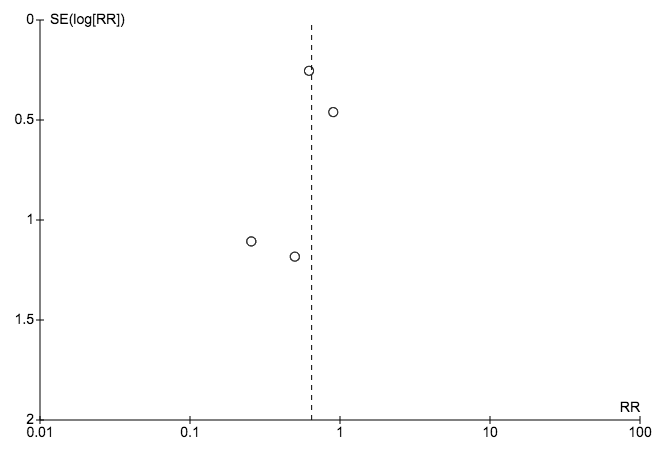


**References**

1. Kyriacos U, Jelsma J, Jordan S. Monitoring vital signs using early warning scoring systems: a review of the literature. J Nurs Manag. 2011;19(3):311-330. [doi: 10.1111/j.1365-2834.2011.01246.x] [Medline: 21507102]
2. Mok WQ, Wang W, Liaw SY. Vital signs monitoring to detect patient deterioration: an integrative literature review. Int J Nurs Pract. 2015;21 Suppl 2:91-98. [doi: 10.1111/ijn.12329] [Medline: 26125576]
3. Ghosh E, Eshelman L, Yang L, Carlson E, Lord B. Description of vital signs data measurement frequency in a medical/surgical unit at a community hospital in United States. Data Brief. 2018;16:612-616. [FREE Full text] [doi: 10.1016/j.dib.2017.11.053] [Medline: 29264378]
4. Manocchia A. Telehealth: enhancing care through technology. R I Med J. 2020;103(1):18-20. [FREE Full text] [Medline: 32013298]
5. Doraiswamy S, Abraham A, Mamtani R, Cheema S. Use of telehealth during the COVID-19 pandemic: scoping review. J Med Internet Res. 2020;22(12):e24087. [FREE Full text] [doi: 10.2196/24087] [Medline: 33147166]
6. The RCP view: Hospital at Home and virtual wards. Royal College of Physicians. URL: https://www.rcp.ac.uk/ policy-and-campaigns/policy-documents/the-rcp-view-hospital-at-home-and-virtual-wards/ [accessed 2025-04-17]
7. Cryer L, Shannon SB, Van Amsterdam M, Leff B. Costs for 'hospital at home' patients were 19 percent lower, with equal or better outcomes compared to similar inpatients. Health Aff (Millwood). 2012;31(6):1237-1243. [doi: 10.1377/hlthaff.2011.1132] [Medline: 22665835]
8. Ko SQ, Chua CMS, Koh SH, Lim YW, Shorey S. Experiences of patients and their caregivers admitted to a hospital-at-home program in Singapore: a descriptive qualitative study. J Gen Intern Med. 2023;38(3):691-698. [FREE Full text] [doi: 10.1007/s11606-022-07765-1] [Medline: 36008593]
9. Whitehead D, Conley J. The next frontier of remote patient monitoring: hospital at home. J Med Internet Res. 2023;25:e42335. [FREE Full text] [doi: 10.2196/42335] [Medline: 36928088]
10. Hospital at Home: Patient Care Model of the Future? Today's Geriatric Medicine. URL: https://www. todaysgeriatricmedicine.com/archive/0313p20.shtml [accessed 2025-04-17]
11. Scott J, Abaraogu UO, Ellis G, Giné-Garriga M, Skelton DA. A systematic review of the physical activity levels of acutely ill older adults in hospital at home settings: an under-researched field. Eur Geriatr Med. 2021;12(2):227-238. [FREE Full text] [doi: 10.1007/s41999-020-00414-y] [Medline: 33058019]
12. Bringing hospital care home: virtual wards and hospital at home for older people. British Geriatrics Society. 2022. URL:

https://www.bgs.org.uk/virtualwards

1. Ko SQ, Tan IS, Cheng SA. The value of vital sign monitoring in detecting clinical deterioration in acute or post-acute medical patients in the community. PROSPERO. 2024. URL: https://www.crd.york.ac.uk/prospero/display_record. php?ID=CRD42023388827 [accessed 2024-04-11]
2. Kohl C, McIntosh E, Unger S, Haddaway N, Kecke S, Schiemann J, et al. Online tools supporting the conduct and reporting of systematic reviews and systematic maps: a case study on CADIMA and review of existing tools. Environ Evid. 2018;7(1). [FREE Full text] [doi: 10.1186/s13750-018-0115-5]
3. Rautiola J, Björklund J, Zelic R, Edgren G, Bottai M, Nilsson M, et al. Risk of postoperative ischemic stroke and myocardial infarction in patients operated for cancer. Ann Surg Oncol. 2024;31(3):1739-1748. [doi: 10.1245/s10434-023-14688-6] [Medline: 38091152]
4. Aranaz-Ostáriz V, Gea-Velázquez De Castro MT, López-Rodríguez-Arias F, San José-Saras D, Vicente-Guijarro J, Pardo-Hernández A, et al. On Behalf Of The Eshmad Director Group And External Advisers. Surgery is in itself a risk factor for the patient. Int J Environ Res Public Health. 2022;19(8):4761. [FREE Full text] [doi: 10.3390/ijerph19084761] [Medline: 35457626]
5. Higgins JPT, Altman DG, Gøtzsche PC, Jüni P, Moher D, Oxman AD, Cochrane Bias Methods Group, et al. Cochrane Statistical Methods Group. The cochrane collaboration's tool for assessing risk of bias in randomised trials. BMJ. 2011;343:d5928. [FREE Full text] [doi: 10.1136/bmj.d5928] [Medline: 22008217]
6. Stang A. Critical evaluation of the newcastle-Ottawa scale for the assessment of the quality of nonrandomized studies in meta-analyses. Eur J Epidemiol. 2010;25(9):603-605. [doi: 10.1007/s10654-010-9491-z] [Medline: 20652370]
7. Murad MH, Sultan S, Haffar S, Bazerbachi F. Methodological quality and synthesis of case series and case reports. BMJ Evid Based Med. 2018;23(2):60-63. [FREE Full text] [doi: 10.1136/bmjebm-2017-110853] [Medline: 29420178]
8. DerSimonian R, Laird N. Meta-analysis in clinical trials. Control Clin Trials. 1986;7(3):177-188. [FREE Full text] [doi: 10.1016/0197-2456(86)90046-2] [Medline: 3802833]
9. Higgins JPT, Thompson SG, Deeks JJ, Altman DG. Measuring inconsistency in meta-analyses. BMJ. 2003;327(7414):557-560. [FREE Full text] [doi: 10.1136/bmj.327.7414.557] [Medline: 12958120]
10. Review Manager (RevMan). Cochrane Collaboration. 2024. URL: https://revman.cochrane.org [accessed 2025-04-17]
11. Prasad M. Introduction to the GRADE tool for rating certainty in evidence and recommendations. Clin Epidemiol Glob Health. 2024;25:101484. [doi: 10.1016/j.cegh.2023.101484]
12. Kimchi A, Aronow HU, Ni YM, Ong MK, Mirocha J, Black JT, et al. Abstract 11007: evaluating the burden of comorbidity on the effect of remote noninvasive tele-monitoring and nurse coaching for patients with heart failure: a secondary analysis of the beat-hf trial. Circulation. 2019;140:A11007. [FREE Full text]
13. Dawson NL, Hull BP, Vijapura P, Dumitrascu AG, Ball CT, Thiemann KM, et al. Home telemonitoring to reduce readmission of high-risk patients: a modified intention-to-treat randomized clinical trial. J Gen Intern Med. 2021;36(11):3395-3401. [FREE Full text] [doi: 10.1007/s11606-020-06589-1] [Medline: 33506388]
14. Indraratna P, Biswas U, McVeigh J, Mamo A, Magdy J, Vickers D, et al. A smartphone-based model of care to support patients with cardiac disease transitioning from hospital to the community (TeleClinical Care): pilot randomized controlled trial. JMIR Mhealth Uhealth. 2022;10(2):e32554. [FREE Full text] [doi: 10.2196/32554] [Medline: 35225819]
15. Noel K, Messina C, Hou W, Schoenfeld E, Kelly G. Tele-transitions of care (TTOC): a 12-month, randomized controlled trial evaluating the use of telehealth to achieve triple aim objectives. BMC Fam Pract. 2020;21(1):27. [FREE Full text] [doi: 10.1186/s12875-020-1094-5] [Medline: 32033535]
16. Olivari ZGS, Giacomelli S, Gubian L, Mancin S, Visentin E, Di Francesco V, et al. The effectiveness of remote monitoring of elderly patients after hospitalisation for heart failure: The renewing health European project. Int J Cardiol. Apr 15, 2018;257(5):137-142. [doi: 10.1016/j.ijcard.2017.10.099] [Medline: 29506685]
17. Ong MK, Romano PS, Edgington S, Aronow HU, Auerbach AD, Black JT, et al. Better Effectiveness After Transition–Heart Failure (BEAT-HF) Research Group. Effectiveness of remote patient monitoring after discharge of hospitalized patients with heart failure: the better effectiveness after transition -- heart failure (BEAT-HF) randomized clinical trial. JAMA Intern Med. 2016;176(3):310-318. [FREE Full text] [doi: 10.1001/jamainternmed.2015.7712] [Medline: 26857383]
18. Rosu RD, Tulai I, Penciu O, Pinkhasova P, Galin I. Heart failure self-management using a mobile web-based telemonitoring system: impact on hospital readmission and quality of life. J Am Coll Cardiol. 2020;75(11):781. [FREE Full text] [doi: 10.1016/s0735-1097(20)31408-x]
19. Schulte P, Olson L, Bruce C. Remote TELEmonitoring and health coaching for patients with acute heart failure: results of the TELE-HC randomized trial. Eur Heart J. 2020;41(Supplement_2). [FREE Full text] [doi: 10.1093/ehjci/ehaa946.1242]
20. Nunes-Ferreira A, Agostinho JR, Rigueira J, Aguiar-Ricardo I, Guimarães T, Santos R, et al. Non-invasive telemonitoring improves outcomes in heart failure with reduced ejection fraction: a study in high-risk patients. ESC Heart Fail. 2020;7(6):3996-4004. [FREE Full text] [doi: 10.1002/ehf2.12999] [Medline: 32949226]
21. Brennan KMD. Harnessing remote patient monitoring technology to improve transitions of care. J Am Geriatr Soc. 2021;69(SUPPL 1):S63. [FREE Full text]
22. Broad JT, Davis C, Bender M, Smith T. Feasibility and acute care utilization outcomes of a post-acute transitional telemonitoring program for underserved heart failure patients. J Card Fail. 2014;20(8):S116. [FREE Full text] [doi: 10.1016/j.cardfail.2014.06.328]
23. Kazankov K, Novelli S, Chatterjee DA, Phillips A, Balaji A, Raja M, et al. Evaluation of CirrhoCare® - a digital health solution for home management of individuals with cirrhosis. J Hepatol. 2023;78(1):123-132. [doi: 10.1016/j.jhep.2022.08.034] [Medline: 36087864]
24. McClinton K, Garganta M, Lato M, Martin L, Kilby S, Kalya A. Hospital readmission rates among disadvantaged heart failure patients enrolled in outreach program. J Card Fail. 2015;21(8):S79. [FREE Full text] [doi: 10.1016/j.cardfail.2015.06.246]
25. Villalba Mora E, Petidier-Torregrossa R, Alonso-Bouzon C, Carnicero-Carreño JA, Rodríguez-Mañas L. Early detection of heart failure exacerbation by telemonitoring in old people. Int J Integr Care. 2015;15(5). [doi: 10.5334/ijic.2173]
26. Prabhakar P, Carter V, Merchant M, Alsalem A, Ku B, Grant A, et al. Heart failure post-discharge telemonitoring in a safety-net hospital reduces 30-day readmissions. J Am Coll Cardiol. 2020;75(11):1058. [FREE Full text] [doi: 10.1016/s0735-1097(20)31685-5]
27. Bennett MK, Shao M, Gorodeski EZ. Home monitoring of heart failure patients at risk for hospital readmission using a novel under-the-mattress piezoelectric sensor: a preliminary single centre experience. J Telemed Telecare. 2017;23(1):60-67. [FREE Full text] [doi: 10.1177/1357633X15618810] [Medline: 26670209]
28. Routledge D, Harrison SJ, Joyce T, Lim S, Montalto M, Todd M, et al. Hospital in the home delivery of supportive care for autologous stem cell transplantation: a novel single centre patient focused approach. Blood. 2021;138(Supplement 1):1834. [FREE Full text] [doi: 10.1182/blood-2021-154290]
29. Power D. An alternative home-based, electronically monitored model of interim care (IC). Ir J Med Sci. 2014;183(suppl 7):S269-S387. [FREE Full text]
30. Berry E. Patient engagement improvement following implementation of a remote monitoring system. In: Acute Heart Failure. 2017. Presented at: Heart Failure 2017 – 4th World Congress on Acute Heart Failure; 2017, April 29 – May 2; Paris, France. URL: https://esc365.escardio.org/presentation/153401
31. Ho K, Novak Lauscher H, Cordeiro J, Hawkins N, Scheuermeyer F, Mitton C, et al. Testing the feasibility of sensor-based home health monitoring (TEC4Home) to support the convalescence of patients with heart failure: pre-post study. JMIR Form Res. 2021;5(6):e24509. [FREE Full text] [doi: 10.2196/24509] [Medline: 34081015]
32. Mitchell JS, McCormick J, Trumbo N, Horne K, Workman J, Lovgren M, et al. Using 30 day remote telemedicine to support heart failure patients following discharge. Heart Lung. 2014;43(4):382. [FREE Full text] [doi: 10.1016/j.hrtlng.2014.06.011]
33. Kargiannakis M, Fitzsimmons DA, Bentley CL, Mountain GA. Does telehealth monitoring identify exacerbations of chronic obstructive pulmonary disease and reduce hospitalisations? An analysis of system data. JMIR Med Inform. 2017;5(1):e8. [FREE Full text] [doi: 10.2196/medinform.6359] [Medline: 28330829]
34. Hsu M. Smart Care: a telecare service for the elder in Taiwan. Gerontechnology. 2014;3(2):107-108. [FREE Full text] [doi: 10.4017/gt.2014.13.02.364.00]
35. Dickinson M, Vos K. Home telehealth done in an integrated disease management program results in substantial cost savings and reduction in healthcare utilization. J Card Fail. 2015;21(8):S78. [FREE Full text] [doi: 10.1016/j.cardfail.2015.06.243]
36. Proctor S, Molloy W, Chai-Coetzer C, Catcheside P, Adams R, Mukherjee S. Under-mattress sleep monitoring to predict readmission risk after COPD exacerbation. SLEEP Adv. 2021;2(Abstract Supplement, Poster). [doi: 10.1093/sleepadvances/zpab014.155]
37. Sandbank AGSB. Abstracts of the 18th Congress of the European Geriatric Medicine Society. Eur Geriatr Med. Dec 05, 2022;13(S1):1-439. [doi: 10.1007/s41999-022-00711-8]
38. Stehlik J, Schmalfuss C, Bozkurt B, Nativi-Nicolau J, Wohlfahrt P, Wegerich S, et al. Continuous wearable monitoring analytics predict heart failure hospitalization: the LINK-HF multicenter study. Circ Heart Fail. 2020;13(3):e006513. [doi: 10.1161/CIRCHEARTFAILURE.119.006513] [Medline: 32093506]
39. Paludo J, Bansal R, Holland AT, Haugen KL, Spychalla MT, McClanahan AL, et al. Pilot implementation of remote patient monitoring program for outpatient management of CAR-T cell therapy. Blood. 2021;138(Supplement 1):568. [FREE Full text] [doi: 10.1182/blood-2021-149103]
40. Leong MQ, Lim CW, Lai YF. Comparison of hospital-at-home models: a systematic review of reviews. BMJ Open. 2021;11(1):e043285. [FREE Full text] [doi: 10.1136/bmjopen-2020-043285] [Medline: 33514582]
41. Patel R, Thornton-Swan TD, Armitage LC, Vollam S, Tarassenko L, Lasserson DS, et al. Remote vital sign monitoring in admission avoidance hospital at home: a systematic review. J Am Med Dir Assoc. 2024;25(8):105080. [FREE Full text] [doi: 10.1016/j.jamda.2024.105080] [Medline: 38908399]
42. Sankey CB, McAvay G, Siner JM, Barsky CL, Chaudhry SI. "Deterioration to Door Time": an exploratory analysis of delays in escalation of care for hospitalized patients. J Gen Intern Med. 2016;31(8):895-900. [FREE Full text] [doi: 10.1007/s11606-016-3654-x] [Medline: 26969311]
43. Leuvan CHV, Mitchell I. Missed opportunities? An observational study of vital sign measurements. Crit Care Resusc. 2008;10(2):111-115. [Medline: 18522524]
44. Bellomo R, Ackerman M, Bailey M, Beale R, Clancy G, Danesh V, et al. Vital Signs to Identify‚ Target‚Assess Level of Care Study (VITAL Care Study) Investigators. A controlled trial of electronic automated advisory vital signs monitoring in general hospital wards. Crit Care Med. 2012;40(8):2349-2361. [doi: 10.1097/CCM.0b013e318255d9a0] [Medline: 22809908]
45. Seshadri DR, Bittel B, Browsky D, Houghtaling P, Drummond CK, Desai M, et al. Accuracy of the apple watch 4 to measure heart rate in patients with atrial fibrillation. IEEE J Transl Eng Health Med. 2020;8:2700204. [FREE Full text] [doi: 10.1109/JTEHM.2019.2950397] [Medline: 32128290]
46. Welch J, Dean J, Hartin J. Using NEWS2: an essential component of reliable clinical assessment. Clin Med (Lond). 2022;22(6):509-513. [FREE Full text] [doi: 10.7861/clinmed.2022-0435] [Medline: 36427875]
47. Jacobsen M, Dembek TA, Kobbe G, Gaidzik PW, Heinemann L. Noninvasive continuous monitoring of vital signs with wearables: fit for medical use? J Diabetes Sci Technol. 2021;15(1):34-43. [FREE Full text] [doi: 10.1177/1932296820904947] [Medline: 32063034]
48. Holloway WJ. Management of sepsis in the elderly. Am J Med. 1986;80(6B):143-148. [doi: 10.1016/0002-9343(86)90492-4] [Medline: 3728524]
